# CRF1 and ACTH inhibitors are a promising approach to treat obesity and leptin and insulin resistance

**DOI:** 10.3389/fendo.2025.1647028

**Published:** 2025-09-25

**Authors:** Patricio H. Contreras, Henrik Falhammar

**Affiliations:** ^1^ Reproductive Health Research Institute, Santiago, Chile; ^2^ Department of Endocrinology, Karolinska University Hospital, Stockholm, Sweden; ^3^ Department of Molecular Medicine, Karolinska Institutet, Stockholm, Sweden

**Keywords:** crinecerfont, atumelnant, leptin resistance, insulin resistance, ACTH inhibitors, hypercortisolism, CRF1 inhibitors

## Abstract

**Evidence synthesis:**

Obesity is a state of subtle hypercortisolism accompanied by leptin and insulin resistance. Serum total cortisol concentration is normal or slightly subnormal in obese subjects. However, they have high cortisol production rates. Reduced concentrations of serum cortisol-binding globulin caused by hyperinsulinemia explain this paradox. Elevated free cortisol concentrations act on the adipose cells of these patients due to excess adrenal cortisol secretion, as well as to high adipocyte expression of 11-beta-hydroxysteroid dehydrogenase type 1 (11β-HSD1). CRF1 and ACTH blockers (such as crinecerfont and atumelnant, respectively) may replace leptin action on the adrenal axis by reducing adrenal cortisol excess in individuals with obesity.

**Context:**

Individuals with obesity experience a subtle hypercortisolism secondary to leptin resistance. Weight loss is commonly followed by a weight rebound, likely provoked by residual leptin resistance. Since leptin inhibits the adrenal axis, leptin resistance induces a hypersecretion of cortisol, promoting chronic, pathological lipolysis of white adipose tissue. The latter situation leads to lean organ steatosis, muscle and liver insulin resistance, and β-cell apoptosis. So, there is an urgent need to restore adrenal inhibition in obese individuals.

**Evidence acquisition:**

We searched PubMed articles and included them if relevant.

**Conclusions:**

Individuals with obesity and high levels of adipose insulin resistance may benefit from CRF1/ACTH inhibitors, reducing ACTH secretion or its action. A reduction in their adipose resistance index may lead to diminished lean organ steatosis and reduced appetite due to a decrease in orexigenic signals, mainly free cortisol and insulin.

## Introduction

Overweight and obesity are increasingly prevalent in many countries (1). This positive energy balance causes highly prevalent morbidities. How to efficiently attack overweight and obesity is still an open question. In our opinion, the high palatability of processed foods, plus their easy availability and affordable price, makes them addictive.

Obesity is the most frequently acquired cause of the twin epidemics: leptin and insulin resistance (LR and IR, respectively). We believe that LR precedes IR by causing pathological lipolysis of fat tissue, which raises the serum concentrations of free fatty acids (FFA). These, in excess, cause steatosis of the liver, muscle, and pancreatic β-cells (2, 3). The reason explaining why a leptin deficit (aleptinemia or hypoleptinemia) and a leptin excess are both associated with adipocyte insulin resistance (AIR) is simple: a cortisol excess accompanies both situations, as leptin can no longer inhibit the adrenal axis. High serum free cortisol concentrations will promote pathological lipolysis of white adipose tissue (WAT). Similarly, mice unable to produce or recognize leptin (Ob/Ob mice and Db/Db mice, respectively) are not only obese but also exhibit IR and adrenal hyperplasia, along with elevated corticosterone concentrations (4).

This subtle hypercortisolism in obese subjects causes pathological lipolysis (independent of caloric needs), with elevated serum free fatty acids (FFA), leading to steatosis of the liver (increased gluconeogenesis), muscle (reduced insulin-mediated glucose uptake), and pancreatic β-cells (increased apoptosis). Therefore, inhibiting lipolysis could be a key therapeutic target, given that it allows for the downregulation of cortisol secretion. We can achieve this by either blocking the activity of hypothalamic CRF with crinecerfont (a CRF receptor type 1 blocker) or by inhibiting ACTH activity at the adrenocortical level with atumelnant, a melanocortin type 2 receptor (MC2R) antagonist. These drugs, aimed at improving the management of congenital adrenal hyperplasia (CAH) in children and adults, may offer a promising new approach to obesity management.

If these drugs block the appearance of lean tissue steatosis, IR, and dysglycemic states in obese patients, the benefit would be enormous since we could block the development of type 2 diabetes and coronary morbidity. This mini-review aims to examine this hypothesis in detail.

## Thomas Addison and Charles Édouard Brown-Séquard

In 1855, Addison described the disease bearing his name, characterized by the primary destruction of the adrenals through atrophy or tuberculous involvement, translating into weight loss, skin hyperpigmentation, and generalized weakness (5). Addison postulated that the adrenals produce some internal secretions essential to sustain life. This publication piqued the interest of the physiologist Brown-Séquard, who demonstrated in 1856 that bilateral adrenalectomy caused the death of guinea pigs. In contrast, the unilateral procedure allowed them to survive, supporting Addison’s hypothesis.

However, it was not until 1936 that Kendall isolated this substance, which he named cortisol. Cortisol hypersecretion in Cushing syndrome or the use of corticosteroids to ameliorate inflammation are associated with obesity. Patients with Addison disease are skinny, prone to hypotension and hypoglycemia, and also highly insulin-sensitive. Contrariwise, those affected with Cushing syndrome are obese, prone to hypertension and hyperglycemia, and also highly IR. Excess cortisol mediates the development of acquired IR in Cushing syndrome (6).

## Is LR-induced hypercortisolism the key to the obesity rebound phenomenon?

There is a strong tendency to regain weight following any successful treatment. Is the remaining LR the key factor inducing this phenomenon by perpetuating unnecessary hunger? While LR cannot be measured directly, an easily obtainable proxy exists. The adipose IR index (fasting FFA *fasting insulin) reflects LR. So, an elevated adipose IR index after weight loss (residual LR) may indicate a high risk of an obesity rebound. In that case, a pharmacologically-induced reduction of AIR would be a key target to prevent an obesity rebound.

We postulate that an LR-induced, subtle hypercortisolism accompanying obesity exacerbates non-physiological lipolysis of WAT by producing AIR, which in turn causes steatosis of lean tissues and hepatic and muscular IR. On the other hand, steatosis of the pancreatic β-cell induces its apoptosis, limiting insulin secretion. These elements pave the road to type 2 diabetes! Additionally, if LR persists after weight loss, the patient may experience unnecessary hunger, leading to an obesity rebound.

## Physiology of lipolysis

Insulin is the antilipolytic hormone par excellence (7–9). Activin E is an antilipolytic hepatokine produced by the liver in response to high serum FFA (10). Lipolytic hormones are catecholamines (epinephrine and norepinephrine) or natriuretic peptides (7). Cortisol and growth hormone are strong lipolytic promoters that enhance the response to lipolytic hormones (7). Several lipolysis actors play crucial roles in regulating fat metabolism, as detailed in **Table 1**.

**Table 1:** Actors playing active roles on lipolysis.

Antilipolytic hormones	Lipolytic hormones	Lipolytic promoters	Adenyl-cyclase key enzyme	Guanylyl-cyclase key enzyme
*INSULIN ACTIVIN E*	*CATECHOLAMINES (CA*) *Epinephrine Norepinephrine*	CORTISOL	*Stimulated by high-dose CA, by activating* β1 *and* β2 *adrenoreceptors*	*Stimulated by Natriuretic Peptides*
		GROWTH HORMONE	*Inhibited by low-dose CA, by activating* α*2 adrenoreceptors*	
			C-AMP	G-AMP
	*NATRIURETIC PEPTIDES*	*They sensitize* β1 *and* β2 *adrenoreceptors*	*Phosphokinase A phosphorylates Hormone-Sensitive Lipase*	*Phosphokinase G phosphorylates Hormone-Sensitive Lipase*

Insulin is the key antilipolytic hormone. Activin E, a hepatokine, is a recently found anti-lipolytic hormone secreted in response to high serum free fatty acids. Adipose insulin resistance implies that insulin can no longer inhibit lipolysis due to a leptin resistance-induced adrenal cortisol hypersecretion. Catecholamines and natriuretic peptides are the direct lipolytic hormones. Cortisol and growth hormone are lipolytic promoters (they enhance the cellular response to lipolytic hormones). Cyclic AMP stimulates Phosphokinase A, and cyclic GMP stimulates Phosphokinase G. Both phosphokinases phosphorylate Perilipin-1 and Hormone-Sensitive Lipase (diacylglycerol-kinase). Anti-glucocorticoid drugs like mifepristone strongly oppose cortisol-induced lipolysis.

Physiological lipolysis occurs to meet energy demands. Lipolysis is pathological when it does not happen to meet an energy requirement. Pathological lipolysis leads to IR, whereas physiological lipolysis does not. As an example of physiological lipolysis, we recall the *second half of nighttime sleep*, when liver glycogen stores become depleted and the body removes glycerol from the WAT’s adipocytes to allow for liver neoglucogenesis, generating glucose to feed the brain. Additionally, during periods of *stress*, the secretion of catecholamines and cortisol increases, and the increased lipolysis provides extra energy. Finally, *aerobic exercise* is another example of physiological lipolysis, associated with increased serum concentrations of catecholamines and natriuretic factors.

The leading causes of pathological lipolysis are common obesity and inflammation (9). Lipolysis does not respond in these cases to an energy demand but to an LR-induced cortisol hypersecretion. These conditions are associated with the triad of LR, lean organ steatosis, and IR (liver and muscle). Lipoatrophic diabetes is a much rarer condition with an extreme form of pathological lipolysis (2). WAT tissue in this condition is prone to cortisol-promoted lipolysis, leading to a severe triglyceride depletion in adipose cells and secondary hypoleptinemia, which further exacerbates cortisol-promoted lipolysis. A rarer yet pathological form of lipolysis is *congenital* aleptinemia, in which adipose cells are overloaded with stored triglycerides but are unable to secrete leptin, leading to abnormal hunger. The pathological lipolysis in this condition is secondary to a cortisol excess, leading to lean organ steatosis and IR. Affected patients, however, are leptin-sensitive and lose weight when treated with recombinant leptin.

LR causes the appearance of pathological lipolysis, removing nutrients (glycerol and FFA) from the WAT in response to excess cortisol secretion. Glycerol promotes liver gluconeogenesis, and re-esterified FFA will deposit as triglycerides in lean tissues (such as the liver and muscle) and pancreatic β-cells. Thus, steatosis develops in these lean tissues, accompanied by an active liver gluconeogenesis due to hepatic IR (despite having serum insulin levels usually able to suppress it), decreased insulin-mediated glucose uptake in muscle (muscle IR), and β-cell apoptosis. Likely, the remaining LR is the key factor inducing this phenomenon by perpetuating unnecessary hunger. While LR cannot be measured directly, an easily obtainable proxy exists: The AIR index (fasting FFA *fasting insulin) reflects LR. So, an elevated AIR index after weight loss may indicate a high risk of an obesity rebound. In that case, a pharmacologically-induced reduction of AIR would be a key target to prevent an obesity rebound.

## Generalized congenital lipoatrophy, an experiment of nature revealing an anti-glucocorticoid-suppressible, extreme adipose IR

Berardinelli described this syndrome (Berardinelli-Seip syndrome) in Brazil in 1954 (11). It carries severe body fat scarcity, profound hypoleptinemia, high hyperinsulinemia, high triglyceride levels, and subnormal HDL cholesterol levels. The condition leads to a very complex diabetes that is untreatable with conventional therapies. It has a full-blown IR and severe steatosis of lean tissues. Recombinant leptin therapy reduces IR and ectopic fat deposits, particularly in the liver. However, this drug is hard to access, so only a minority of patients will benefit from its existence.

A published case report illustrates generalized congenital lipoatrophy (2). In 1986, we received a 16-year-old girl, the daughter of consanguineous parents, with severe lipoatrophic diabetes unresponsive to usual therapies. The physiology of the adipocyte was in its infancy, given that leptin’s existence was unknown until 1994. However, we found in the literature that cortisol played a crucial role as a promoter of lipolysis (12), so we hypothesized that lipoatrophy was a consequence of unrestrained cortisol-induced lipolysis. We speculated that cortisol blockade with mifepristone might reverse the ravages of the disease in our patient (severe eruptive xanthomatosis, hypertriglyceridemia of 7,400 mg/dL, hyperinsulinemia above 400 mU/L, and a fasting glycemia around 200–250 mg/dL).

Not having another therapeutic alternative, we offered the patient and her family the experimental approach of blocking the action of cortisol with 200 mg of mifepristone three times a day (the same dose we had already used in a patient with adrenocortical cancer) for nine weeks. As expected, the patient developed a compensatory hypercortisolism secondary to cortisol receptor blockade, so we decided to add ketoconazole 800 mg daily (a cortisol synthesis blocker) for one week to reduce this compensatory hypercortisolism before returning to use mifepristone alone for an additional two weeks. This therapeutic addition accentuated the impressive positive metabolic changes, reinforcing our initial hypothesis. Clinical and biochemical results were spectacular. Eruptive xanthomas and severe acanthosis nigricans disappeared, and the patient gained seven kilograms of weight, supposedly due to fat deposition, along with impressive drops in insulinemia and triglyceridemia and minor improvements in fasting serum glucose levels. We decided to remove her adrenals and expose her WAT to the lowest possible cortisol concentrations. The results of her adrenalectomy (1988) have been more than acceptable from the metabolic point of view. She has experienced weight gain over the years (currently, she is slightly overweight), and she is one of only two patients with generalized lipoatrophy worldwide who have managed to get pregnant twice with one live child (13).

Our findings on the effects of adrenalectomy in human lipoatrophic diabetes were replicated fourteen years later by Haluzik et al. in a mouse model of lipoatrophic diabetes (14). By limiting adipose tissue exposure to exogenous cortisol in our patient, who received hydrocortisone replacements of 15 mg daily (10 mg in the morning and 5 mg in the evening), we have maintained her life and prevented eruptive xanthomas, albeit with high insulin requirements. The case was formally published in 2022 when we could finally interpret the facts in light of the knowledge accumulated since 1988 (2). Subsequently, we analyzed the beneficial effects of adrenalectomy in humans and a murine model affected by lipoatrophic diabetes (3). Generalized congenital lipoatrophy is a striking example of full-blown AIR, likely secondary to the associated hypoleptinemia, which responds remarkably to mifepristone alone or in combination with ketoconazole (2, 3). In this disease, pharmacological blockade of the adrenal axis with the new oral, non-peptide inhibitors of the axis (crinecerfont and atumelnant) could have a significant therapeutic effect. In other words, recombinant CRF1 and ACTH inhibitors should similarly restrain the disinhibited adrenal axis.

The NIH group led by L. Niemann showed in 2021 that the daily use of mifepristone, 200 mg daily in four doses (a third of the dose we used in our patient), in individuals with obesity and dysglycemia with IR showed an improvement in their adipocyte IR, which fell by 31.2% (15). There was a non-significant drop in hepatic IR and no improvement in muscle IR. Consequently, mifepristone relieves adipocyte IR both in conditions of “triglyceride-replete adipocyte” (obesity) and in conditions of “empty adipocyte” (generalized congenital lipoatrophy), reinforcing our position: both the non-reading of leptin (LR) as well as its deficiency will cause non-physiological lipolysis.

## Obesity is a state of subtle hypercortisolism

In 1932, Cushing described this disease, which produces a grotesque form of central obesity, high blood pressure, dyslipidemia, IR, and dysglycemia (16). This historic milestone is crucial because central obesity is reminiscent of Cushing syndrome but of a lesser magnitude. In 1988, Reaven described the Insulin Resistance Syndrome (17). The most frequent cause of IR, by far, is abdominal obesity, which shares with Cushing syndrome high blood pressure, dyslipidemia, and dysglycemia. This fact led many researchers to speculate that abdominal obesity contained a hidden hypercortisolism. However, serum cortisol concentrations are normal or slightly subnormal in people with obesity. Paradoxically, cortisol production rates are high in these individuals. This discrepancy arises from the low concentrations of cortisol-binding globulin (CBG) in these patients, secondary to hyperinsulinemia (18).

In people with obesity, high concentrations of free cortisol act on adipocytes for two reasons. Firstly, because the adrenal cortex produces an excess of cortisol (by LR) and secondly, because of an elevated adipocyte expression of the enzyme 11-beta-hydroxysteroid-dehydrogenase type 1 (11b-HSD1) (19) that reactivates extracellular cortisone (without lipolytic action) to cortisol, a key promoter of lipolysis (this enzyme co-localizes with the cortisol receptor within the fat cell). Therefore, people with obesity experience excessive lipolysis, not in response to physiological energy needs but as a consequence of greater exposure of the adipose cell to cortisol.

This pathological lipolysis causes steatosis of lean tissues (liver, muscle, and pancreatic β-cells), the emergence of hepatic and muscular IR, and b-cells apoptosis. Obesity then produces hyperleptinemia with LR. Excess cortisol secretion occurs by decreased leptin-mediated inhibition of the adrenal axis. This cortisol excess increases with the induction of 11β-HSD1 triggered by high cortisol and leptin concentrations. The increased entry of cortisol into the adipose cell causes AIR (a direct reflection of LR), which translates into higher circulating concentrations of FFA, stimulating the ectopic deposition of triglycerides in the liver and muscle, triggering hepatic and muscular IR. Finally, the triglyceride buildup in the insulin-producing β-cells triggers their programmed cell death (20). While AIR is secondary to increased adrenal cortisol secretion, hepatic and muscular IR are secondary to steatosis in these two lean tissues.

By reducing the excessive exposure of adipocytes to cortisol in patients with obesity, there would be no pathological lipolysis, no IR in adipocytes, hepatocytes, or myocytes, and no β-cells apoptosis. In other words, LR would not express itself as AIR, thus blocking the emergence of metabolic derangement in patients with obesity. Only two potentially therapeutic alternatives exist: 11β-HSD inhibitors (in development) or adrenal axis inhibitors (crinecerfont and atumelnant). The CRF1/ACTH inhibitor-mediated restraint of lipolysis may ultimately lead to a reduction in lean tissue steatosis.

## Development of the non-peptide blockers of the adrenal axis: crinecerfont and atulmelnant

ACTH blockers can either inhibit the pituitary secretion of ACTH (via CRF1 receptor blockade) or its action at the adrenocortical level. A 2016 study on eight patients with CAH due to 21-hydroxylase deficiency profiled the effects of a single dose of a CRF1 antagonist at bedtime. This maneuver delayed and attenuated the morning rise of ACTH and 17-hydroxyprogesterone (21). This study investigated the use of CRF1 antagonists to reduce androgen overproduction by employing physiological glucocorticoid replacement doses. The race winner was crinecerfont, which showed highly favorable results in children (22) and adults (23). Crinecerfont has a non-peptide structure and requires two oral doses (100 mg every 12 hours in adults). These two successful phase 3 studies were published in the New England Journal of Medicine and led to the approval of this drug by the FDA in December 2024 (24). Crinecerfont is therefore an adjunctive treatment for controlling androgens in adults and pediatric populations with CAH, starting at 4 years of age.

Atumelnant is a competitor in the markets for CAH and Cushing disease. It is an oral, non-peptide blocker that inhibits the action of ACTH at the adrenal cortex by blocking the MC2R. A nightly dose of 80 mg is effective for both CAH and ACTH-dependent Cushing syndrome. A Phase 3 study is expected to begin recruiting patients this year. Both crinecerfont and atumelnant enable physiological glucocorticoid replacement to lower adrenal androgen levels in patients with CAH (22–26).

## Blocking the non-physiological lipolysis of obesity with adrenal axis inhibitors: expected consequences 

**Figure 1:**
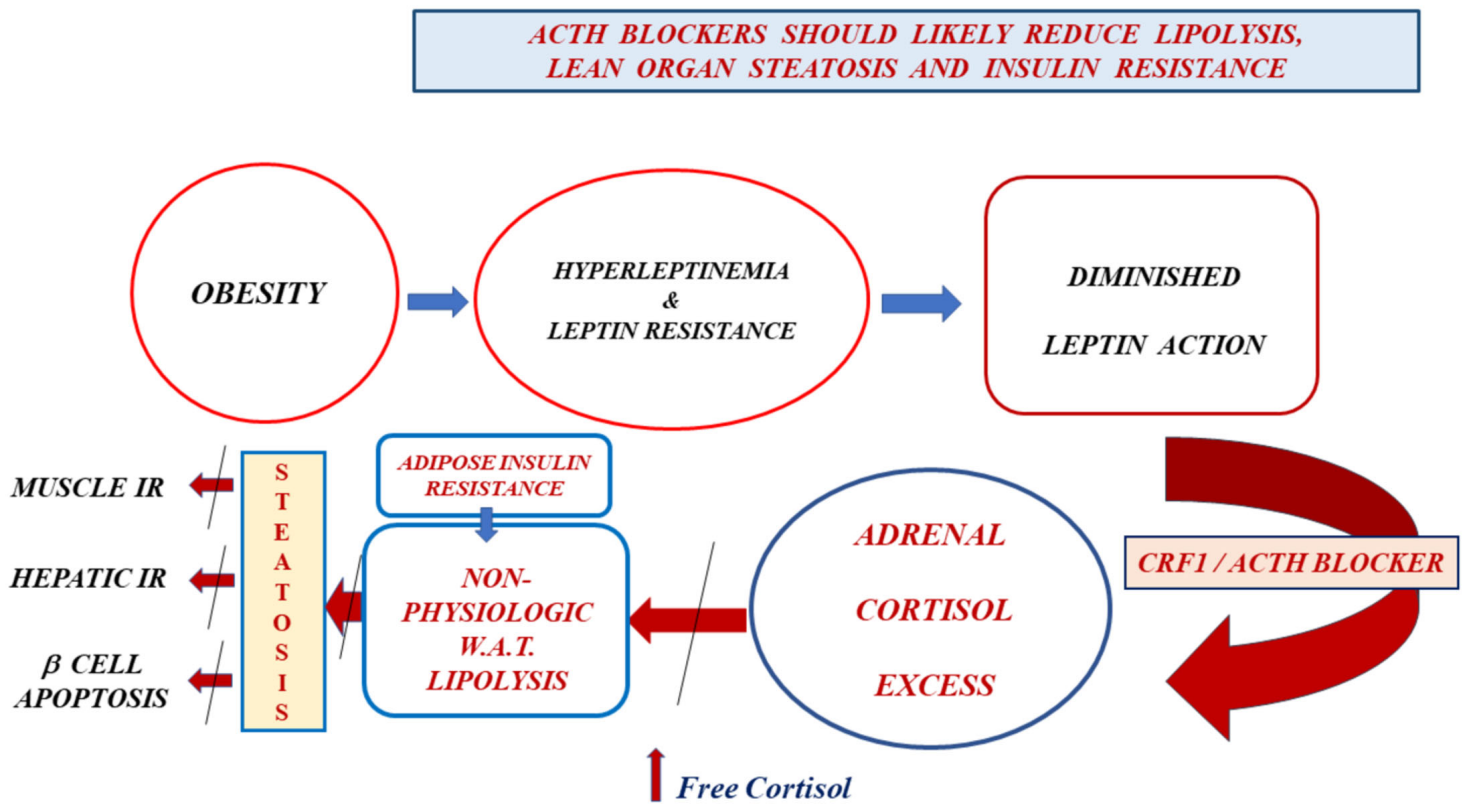
Obesity leads to hyperleptinemia and, consequently, to leptin resistance. The diminished leptin action reduces the adrenal restraint exerted by this hormone secreted by adipocytes. We expect, as a natural consequence of this, a rise in serum concentrations of CRF, ACTH, and cortisol. Even though serum total cortisol concentrations tend to be lower than usual in people with obesity, the free fraction of this hormone increases due to hyperinsulinemia-induced depressed cortisol-binding globulin concentrations. Cortisol excess promotes pathological lipolysis in white adipose tissue (not occurring in response to an energetic need). Adipocyte insulin resistance is a direct consequence of leptin resistance. A chronic excess of free fatty acids in serum leads to steatosis of lean organs, resulting in muscle insulin resistance (low insulin-mediated glucose uptake), liver insulin resistance (elevated liver glucose output), and β-cell apoptosis. CRF1 and ACTH blockers in individuals with obesity and leptin resistance should replace the normal leptin restraint of the adrenal axis that is lost in leptin-resistant subjects, thereby canceling cortisol excess, pathological lipolysis, lean organ steatosis, muscle and liver insulin resistance, and β-cell apoptosis.

We propose testing ACTH inhibitors (crinecerfont and atumelnant) to replace the lost leptin-mediated adrenal axis restraint in patients with either LR (obesity, prediabetes, diabetes type 2, fatty liver disease) or reduced leptin concentrations (aleptinemia, generalized lipodystrophies). All the above-mentioned pathologies are associated with AIR brought about by a reduced leptin action-mediated cortisol excess. Therefore, both a crinecerfont-mediated serum ACTH fall and an atumelnant-MR2C antagonism on the adrenal cortex should reduce the underlying hypercortisolism (**Figure 1**). Additionally, we may oppose this subtle hypercortisolism with either glucocorticoid antagonists such as mifepristone (2, 3, 15) or steroidogenesis inhibitors such as ketoconazole (2, 27). However, both of those alternatives will face an ACTH-override phenomenon (2, 27), with diminishing drug activity over time. In contrast, the ACTH-override phenomenon should not occur when using these ACTH antagonists, given their mechanisms of action (CRF1 antagonism and MC2R antagonism, respectively). Their action may block the pathological sequence previously discussed. Additionally, β-cell apoptosis should decrease due to the decrease in serum FFA. Additionally, a reduction in serum insulin and free cortisol concentrations should decrease appetite, as these are orexigenic. Moreover, there would be less lipogenesis in adipose tissue, with lower leptin concentrations due to a gradual reduction in triglycerides stored in adipocytes. Finally, the potential reversal of steatosis in lean tissues with ACTH blockers may solve the serious problem of fatty liver, which is highly prevalent and most likely the most frequent cause of liver cirrhosis.

We must keep in mind that crinecerfont and atumelnant exert differential effects on ACTH secretion and action. While crinecerfont use is associated with a clear and consistent reduction in serum ACTH concentrations, atumelnant use in patients with CAH did not disclose a clear pattern of serum ACTH change (26). Both ACTH inhibitors reduce adrenocortical secretions through different mechanisms. Crinecerfont may lessen the secretion of not only ACTH, but also α-MSH (a potent anorexigenic hormone acting on the hypothalamus by acting on melanocortin 4 receptors [MC4R]). But, simultaneously, crinecerfont should lead to reduced circulating free cortisol and insulin, two potent orexigenic hormones. So, a final balance of all these hormonal changes could still be a diminished appetite. Atumelnant, on the other hand, is less likely to be a potential inhibitor of α-MSH. However, it should also reduce circulating free cortisol and insulin, leading to reduced hunger.

## Discussion and conclusions

Obesity and related pathologies are among the biggest challenges facing modern Medicine, as they are responsible for a large portion of morbidity affecting people, from childhood to old age. Similarly, they consume a growing portion of the resources that societies allocate to healthcare. The problem lies in the fact that we do not know yet how to prevent and treat these diseases cost-effectively.

Mice devoid of leptin-mediated adrenal restraint (Ob/Ob and Db/Db mice) become obese and exhibit IR, adrenal hyperplasia, and elevated corticosterone levels (4). These observations support that LR and leptin reduction lead to excessive glucocorticoid secretion in the adrenal glands.

Obese individuals, even after losing weight, generally do not achieve a normal percentage of body fat. If so, they may retain some degree of hyperleptinemia and residual LR, with elevated AIR and inappropriate hunger leading to weight rebound. Therefore, they may need to lower their AIR to prevent this rebound. Clinical researchers should test ACTH inhibitors in individuals with abdominal obesity who exhibit an elevated AIR index. If these inhibitors significantly decrease AIR, they may also reduce lean organ steatosis, thereby diminishing hepatic and muscle IR and minimizing β-cell apoptosis. These changes should reduce serum glucose and insulin concentrations. Lower insulin and free cortisol concentrations in the serum should decrease appetite, as both hormones are potent stimulators of hunger. Lower insulin and free cortisol concentrations may also reduce adipocyte lipogenesis, decreasing leptin secretion.

The strength of this hypothesis lies in its straightforward testability using crinecerfont in obese individuals with AIR. Researchers should up-titrate carefully the appropriate dosage of ACTH inhibitors to reduce AIR while minimizing the risk of adrenal insufficiency.
