# Correction: CRF1 and ACTH inhibitors are a promising approach to treat obesity and leptin and insulin resistance

**DOI:** 10.3389/fendo.2025.1715691

**Published:** 2025-10-06

**Authors:** Patricio H. Contreras, Henrik Falhammar

**Affiliations:** ^1^ Reproductive Health Research Institute, Santiago, Chile; ^2^ Department of Endocrinology, Karolinska University Hospital, Stockholm, Sweden; ^3^ Department of Molecular Medicine, Karolinska Institutet, Stockholm, Sweden

**Keywords:** crinecerfont, atumelnant, leptin resistance, insulin resistance, ACTH inhibitors, hypercortisolism, CRF1 inhibitors

“Affiliation Reproductive Health Research Institute, Santiago, Chile” was erroneously given as “Reproductive Endocrinology Unit, Santiago, Chile”.

The original version of this article has been updated.

